# Supplementary material for: Increased scalability and sequencing quality of an epigenetic age prediction assay
Source: PLoS One. 2024 May 14;19(5):e0297006. doi: 10.1371/journal.pone.0297006 (PMC11093300; doi:10.1371/journal.pone.0297006)
Supplement: S1 File — (DOCX) [file pone.0297006.s001.docx]

Supplementary to Increased scalability and sequencing quality of an epigenetic age prediction assay

Benjamin Mayne, David Chandler, Christopher Noune, Tom Espinoza, David Roberts, Chloe Anderson, and Oliver Berry

**Contents**

| **Section** | **Page** |
| --- | --- |
| **Supplementary Methods** | **2** |
| Primer Sequences | 2 |
| Multiplex PCR Master mix | 3 |
| Multiplex PCR Cycling Conditions | 3 |
| Barcoding Master mix | 3 |
| Barcoding Cycling Conditions | 3 |
| PCR Product Clean up protocol | 4 |
| **Supplementary Results** | **5** |
| Tapestation Electropherogram | 5 |
| Reagent Comparison | 6 |

**Supplementary Methods**

**Supplementary Table 1.** List of primers used in this study. The lower-case bases represent the nextera-overhang sequence for the barcoding reaction.

| **Primer Number** | **Forward Primer Sequence** | **Reverse Primer Sequence** |
| --- | --- | --- |
| 1 | tcgtcggcagcgtcagatgtgtataagagacagTCACAACCNAAAAAAACTTCCC | gtctcgtgggctcggagatgtgtataagagacagGTTTTGGGNGTTATATGAAATTAAG |
| 2 | tcgtcggcagcgtcagatgtgtataagagacagGTNGGAGATTTGATGTAAGTTG | gtctcgtgggctcggagatgtgtataagagacagTAAAATAAACCCTATATCAAACCCT |
| 3 | tcgtcggcagcgtcagatgtgtataagagacagAATATTTTTGGTAAGGATGTTGTTG | gtctcgtgggctcggagatgtgtataagagacagTATTAATTTTCTTAACATACATCTCCC |
| 4 | tcgtcggcagcgtcagatgtgtataagagacagACCTCTTATTCTTAAACCAATTACT | gtctcgtgggctcggagatgtgtataagagacagAGATTATGTATTGTTTTAAGGAGAG |
| 5 | tcgtcggcagcgtcagatgtgtataagagacagAGGTTATTATAAGTGGAAATTTGAG | gtctcgtgggctcggagatgtgtataagagacagCCCCCNAATATCTTATCTACC |
| 6 | tcgtcggcagcgtcagatgtgtataagagacagACTATTTTTTTCACAATACCCATTTTC | gtctcgtgggctcggagatgtgtataagagacagGGANGTAATGAGGTTTAGTATTA |
| 7 | tcgtcggcagcgtcagatgtgtataagagacagGAGAATAATGGATAGGTAAGTGT | gtctcgtgggctcggagatgtgtataagagacagCACACCCTCNATATATAATAACT |
| 8 | tcgtcggcagcgtcagatgtgtataagagacagTTATGAATGTTGTGTGATTAGTTTG | gtctcgtgggctcggagatgtgtataagagacagTACATACATAAATAAATCATCCCAC |
| 9 | tcgtcggcagcgtcagatgtgtataagagacagGTGGTAAGNGTTTTTAAGATGAG | gtctcgtgggctcggagatgtgtataagagacagTAACTATATACTATACAATACCACTAC |
| 10 | tcgtcggcagcgtcagatgtgtataagagacagCACTATAACNTCCTTAAAAATCTTC | gtctcgtgggctcggagatgtgtataagagacagTNGTAGTTTGATTTTGGATGATAG |
| 11 | tcgtcggcagcgtcagatgtgtataagagacagATATCCACAACCCCTCCCT | gtctcgtgggctcggagatgtgtataagagacagGAATTGTTAAGTATTTAGAGGATAG |
| 12 | tcgtcggcagcgtcagatgtgtataagagacagCCAAAAAATAACCCACCCCAT | gtctcgtgggctcggagatgtgtataagagacagTGGTNGGTTTTGTGTAGTTTG |
| 13 | tcgtcggcagcgtcagatgtgtataagagacagTGGTTTTTGTAGGAAGAGAATGA | gtctcgtgggctcggagatgtgtataagagacagATTCCCNACTCATTACTACTATA |

**Supplementary Table 2.** Multiplex PCR mater mix.

| **Component** | **Stock Concentration** | **Volume (µL)** |
| --- | --- | --- |
| 4X Platinum™ SuperFi™ U Multiplex PCR Master Mix | 4x | 6.25 |
| Pooled Primers | 0.8µM | 1 |
| Nuclease Free Water | NA | 15.75 |
| DNA | Bisulfite/Enzymatic | 2 |

**Supplementary Table 3.** Cycling conditions for the multiplex PCR.

| **Temperature (°C)** | **Duration (s)** | **Cycles** |
| --- | --- | --- |
| 98 | 30 | 1 |
| 94 | 15 | 35 |
| 55 | 300 |  |
| 72 | 30 |  |
| 72 | 30 | 1 |

**Supplementary Table 4.** Master mix for the barcoding reaction. The Illumina barcodes used in this study are catalogue #20025019.

| **Component** | **Stock Concentration** | **Volume (µL)** |
| --- | --- | --- |
| 4X Platinum™ SuperFi™ U Multiplex PCR Master Mix | 4x | 6.25 |
| Illumina Barcode | Neat, stock | 5 |
| Nuclease Free Water | NA | 8.75 |
| Multiplex PCR Product | NA | 5 |

**Supplementary Table 5.** Cycling conditions for the barcoding reaction.

| **Temperature (°C)** | **Duration (s)** | **Cycles** |
| --- | --- | --- |
| 98 | 30 | 1 |
| 98 | 10 | 8 |
| 60 | 10 |  |
| 72 | 30 |  |

**Clean-up protocol with SPRI bead mix**

The full protocol to make the SPRI Bead mix is available at <https://openwetware.org/wiki/SPRI_bead_mix>

1. Add 65μL (1.3x Volume) of the SPRI DNA Bead Mix to each well for the multiplex PCR cleanup and 30μL (1.0x Volume) for barcoding cleanup.
2. Mix thoroughly by pipetting up and down at least 10 times and allow to incubate at room temperature for 10mins.
3. Place the plate on the magnetic plate and remove supernatant once beads are attached to the sides of the wells.
4. Wash the DNA sample twice with 200μL of 70% Ethanol. Spin the plate and remove any excess ethanol prior to the next step.
5. Resuspend beads in 30μL of low TE buffer (10mM Tris-HCl and 0.1mM EDTA) and incubate at room temperature for 10mins.

**Supplementary Results**


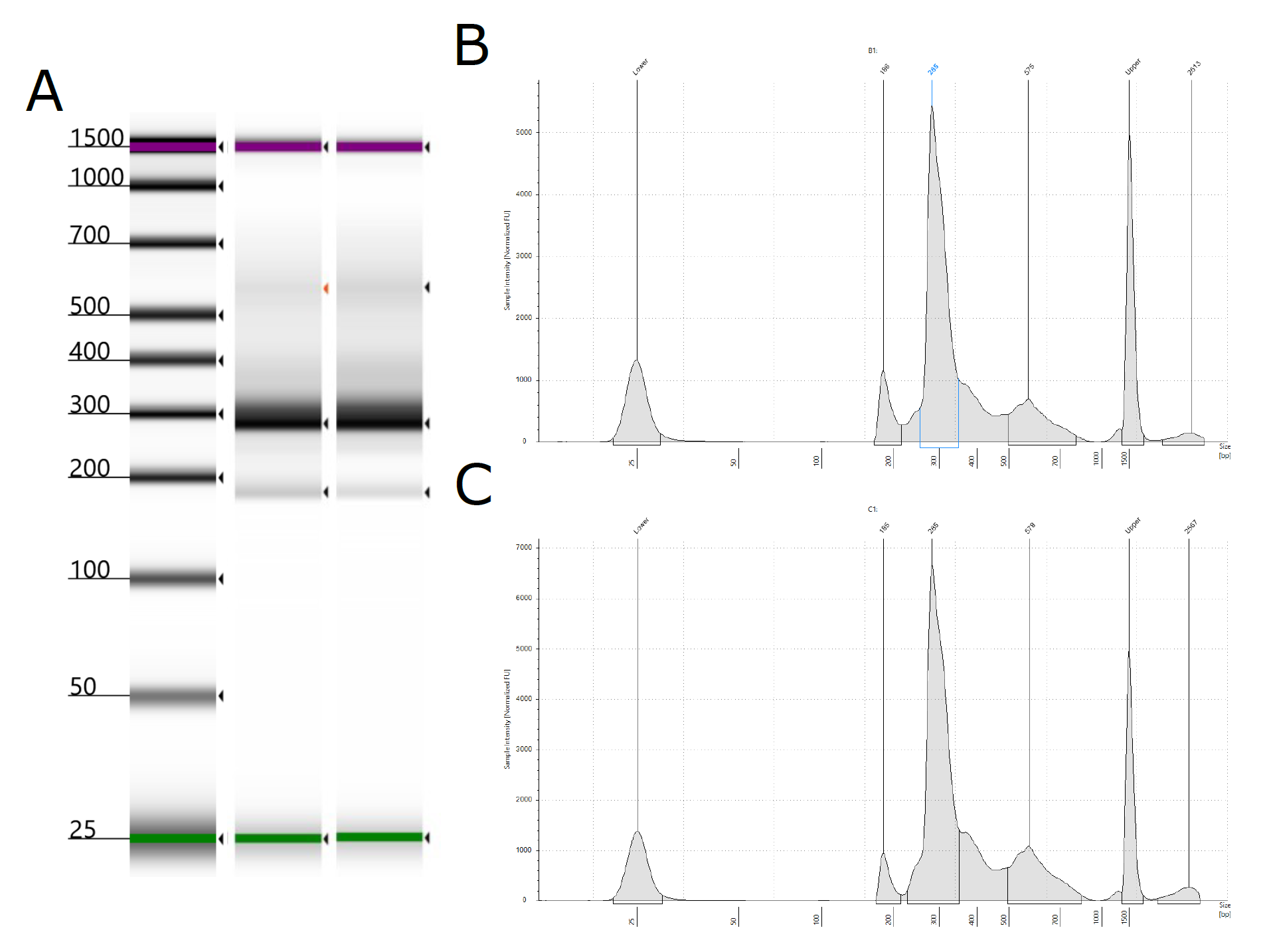


**Supplementary Figure 1.** Library preparation of both bisulfite and enzymatic treated DNA. **A.** Electronic gel photo of both libraries, the first lane is an electronic DNA ladder. Electrograms of **B.** Bisulfite treated and **C.** Enzymatic treated libraries.


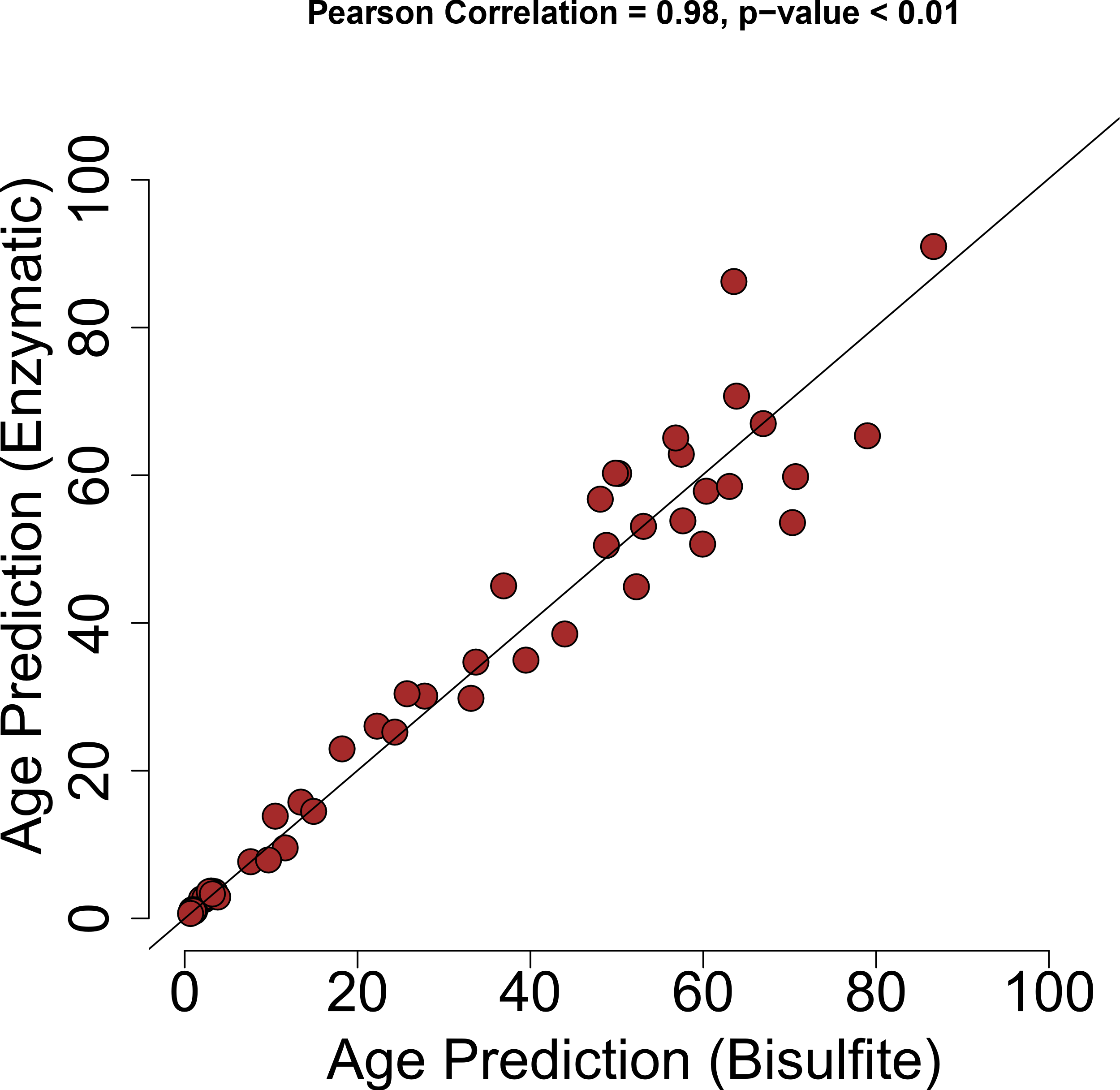


**Supplementary Figure 2.** Correlation between the age prediction using bisulfite compared to enzymatic treatment (Pearson correlation = 0.98, p-value < 0.01).

**Supplementary Table 6.** Comparison of the total number of reagents per step in the assay to age prediction.

| **Reagents** | **Bisulfite** | **Enzymatic** | **In-house PCR master mix** | **Commercial PCR master mix** |
| --- | --- | --- | --- | --- |
| 1 | CT Conversion Reagent | Elution Buffer | 5x Green GoTaq Flexi Buffer | 4X Platinum™ SuperFi™ U Multiplex PCR Master Mix |
| 2 | M-Dissolving Buffer | TET2 Reaction Buffer | GoTaq Hot Start Polymerase | Pooled Primers |
| 3 | M-Dilution Buffer | TET2 Reaction Buffer Supplement | Mg | Nuclease Free Water |
| 4 | M-Wash Buffer | Oxidation Supplement | dNTP |  |
| 5 | M-Binding Buffer | Oxidation Enhancer | Betaine |  |
| 6 | M-Desulphonation Buffer | TET2 | DTT |  |
| 7 | M-Elution Buffer | FE (II) Solution | DMSO |  |
| 8 | Ethanol | Stop Reagent | BSA |  |
| 9 |  | APOBEC | TMAC |  |
| 10 |  | APOBEC Reaction Buffer | Pooled Primers |  |
| 11 |  |  | Water |  |
| **Total Reagents** | **8** | **10** | **11** | **3** |
