# Supplementary figures and images for: Increased scalability and sequencing quality of an epigenetic age prediction assay
Source: PLoS One. 2024 May 14;19(5):e0297006. doi: 10.1371/journal.pone.0297006 (PMC11093300; doi:10.1371/journal.pone.0297006)

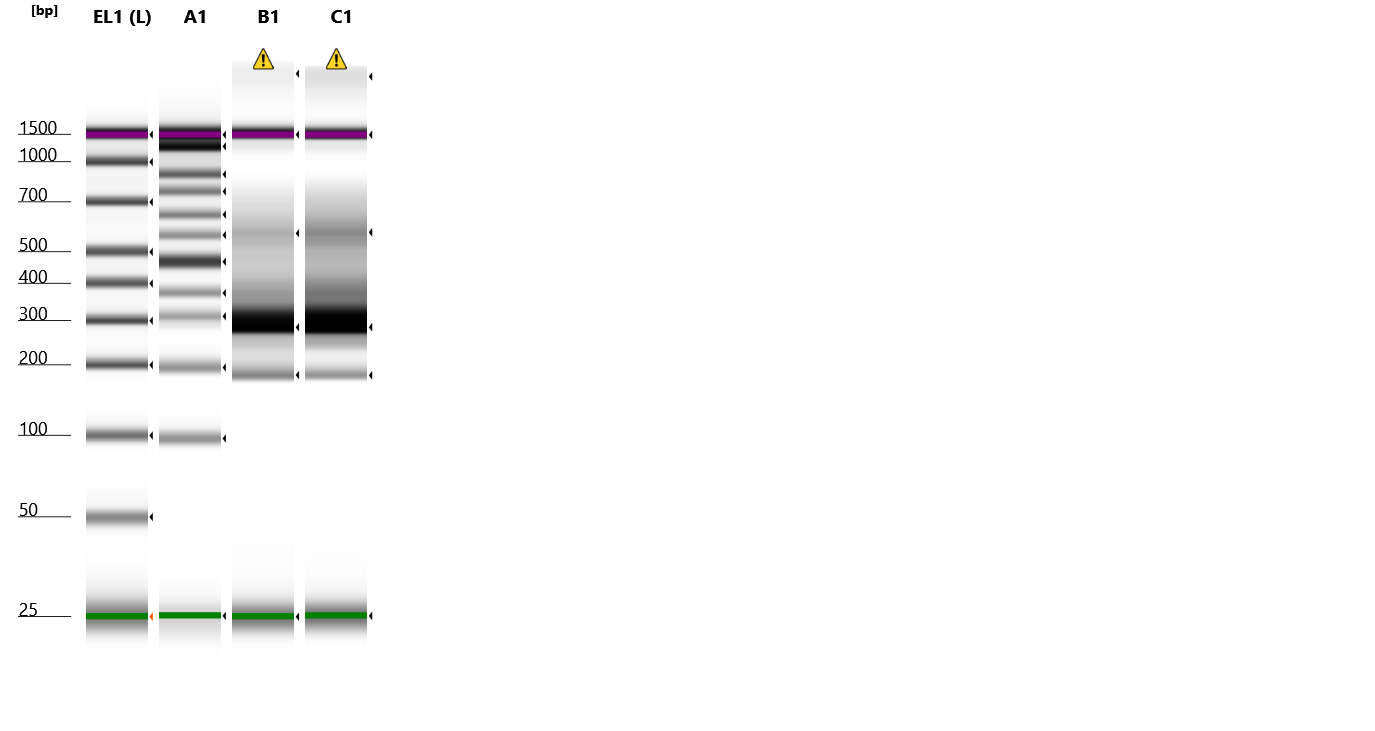

Supplement: S1 Raw image — (PNG) [file pone.0297006.s002.png]
